# Individual-, Family-, Community-, and Policy-Level Impact of a School-Based Cardiovascular Risk Detection Screening Program for Children in Underserved, Rural Areas: The CARDIAC Project

**DOI:** 10.1155/2013/732579

**Published:** 2013-06-05

**Authors:** Lesley Cottrell, Collin John, Emily Murphy, Christa L. Lilly, Susan K. Ritchie, Eloise Elliott, Valerie Minor, William A. Neal

**Affiliations:** ^1^Department of Pediatrics, School of Medicine, West Virginia University, One Medical Drive, P.O. Box 9214, RCBHSC, Morgantown, WV 26506-9214, USA; ^2^The West Virginia University Extension Service, 29 Beechurst Avenue, Morgantown, WV 26506-6031, USA; ^3^Department of Biostatistics, School of Public Health, West Virginia University, P.O. Box 9190, Morgantown, WV 26506-9190, USA; ^4^College of Physical Activity and Sports Sciences, West Virginia University, P.O. Box 6116, Morgantown, WV 26506-6116, USA; ^5^School of Nursing, Alderson Broaddus College, 101 College Hill Drive, Phillipi, WV 26416, USA

## Abstract

The Coronary Artery Risk Detection In Appalachian Communities (CARDIAC) Project has screened more than 80,000 children (10–12 years) for cardiovascular and diabetes risk factors over the past 15 years. Simultaneous referral and intervention efforts have also contributed to the overall program impact. In this study, we examined evidence of programmatic impact in the past decade at the individual, family, community, and policy levels from child screening outcomes, referral rates, participation in subsequent services, and policies that embed the activities of the project as a significant element. Within this period of time, fifth-grade overweight and obesity rates were maintained at a time when rates elsewhere increased. 107 children were referred for additional screening and treatment for probable familial hypercholesterolemia (FH); 82 family members were subsequently screened in family-based screening efforts. 58 grants were distributed throughout the state for community-appropriate obesity intervention. A state wellness policy embedded CARDIAC as the method of assessment and national child cholesterol screening guidelines were impacted by CARDIAC findings. The sustainability and successful impact of this school-based program within a largely underserved, rural Appalachian state are also discussed.

## 1. Introduction 

The prevalence of childhood obesity and health concerns associated with it including insulin resistance, hypertension, and dyslipidemia has steadily increased over the past three decades in the United States and internationally [1–3]. Increased prevalence of these health conditions and attendant media coverage has contributed to increased public awareness and demand for effective detection and treatment [4]. Universal obesity detection, particularly programs that are school based, has received mixed reactions from health care providers, school personnel, parents, and communities [5, 6]. 

 Screening guidelines for comorbidities such as hypercholesterolemia traditionally target only select groups of children based on family history [7] in an effort to limit cost and deter overuse of cholesterol-lowering medication. Current guidelines recommend blood cholesterol screening for all children [8]. 

 Comparison of the impact and efficiency of universal versus targeted screening programs has been rare given the limited number of universal screening programs throughout the United States for children [9, 10]. Comprehensive cardiovascular and metabolic risk detection programs designed to assess comorbidities are also rare. 

 The Coronary Artery Risk Detection In Appalachian Communities (CARDIAC) Project began in 1998 and has screened more than 80,000 fifth-grade students throughout rural, Appalachian West Virginia (WV). Quality improvements related to the program as well as priorities, procedures, and public response have contributed to the success of the program over the past 15 years. Through its surveillance, intervention, research, and educational efforts, The CARDIAC Project has had an impact at the individual, family, community, and policy level. We will review evidence of its programmatic impact at each level and discuss factors that will likely lead to sustainability of programs of this nature. 

## 2. Materials and Methods

### 2.1. Program Purpose

 The primary aim of the CARDIAC Project is to determine the prevalence of children who are obese or overweight and assess associated co-morbidities. The secondary aims of the program include referral of children at risk for developing chronic disease for further evaluation and testing as per guidelines established by the American Academy of Pediatrics and to establish a sustainable statewide health educational program to improve children's health-related knowledge and behaviors.

### 2.2. Setting

The CARDIAC Project was first implemented in 1998 for 5th-grade students enrolled in elementary schools throughout three rural counties in West Virginia (WV). Since that time, the program has expanded to include 53 of the 55 counties in the state. The remaining two counties conducted their own school-based screening programs. The program is conducted within public, and some private, schools throughout the academic year at the beginning of the school day. 

### 2.3. Patient Population

All fifth-grade students are eligible to participate in the CARDIAC Project. Parents of children who participate in CARDIAC are also eligible to receive a free cholesterol screening in their local community. 

### 2.4. Measures

Children participating in the CARDIAC Project receive the following screening services during one assessment period. A health report based on the findings in these areas is sent home to the participant's family between 4 to 6 weeks after screening. 

#### 2.4.1. Body Composition

Children's height and weight are assessed using SECA Road Rod stadiometers and the SECA 840 digital scales (Seca Corp, Hanover, MD, USA) after their shoes, extra clothing, and hats are removed. Body Mass Index (BMI) is then calculated by *EpiInfo* using the following equation: BMI (kg/m^2^) = weight (kg)/height (m)^2^. Age- and gender-specific growth charts are then compared to each child's BMI values to calculate a BMI percentile value [11]. BMI percentiles are then often recoded into four categories for interpretation: underweight (0–4.9th%), healthy weight (5–84.9th%), overweight (85–94.9th%), and obese (≥95th%). 

#### 2.4.2. Blood Pressure

Two assessments of blood pressure are completed using the Welch Allyn Cuff (NY, USA), and if significantly different from one another, a third measurement is conducted. Pressures are adjusted for height, age, and gender to calculate blood pressure percentiles. Percentiles ≥95th percentile are considered abnormal.

#### 2.4.3. Prediabetes

The acanthosis nigricans (AN) marker is characterized as a pigmented rash on the neck or axilla. CARDIAC screening incorporates an exam of the back and base of each child's neck for the marker. Screening personnel are trained on ways to detect the marker during summer training sessions and report either the presence or absence on a screening form.

#### 2.4.4. Lipid Analyses

Trained volunteer phlebotomists from local communities collect the fasting blood sample from each participating child. All samples are forwarded and analyzed by a commercial reference laboratory or local hospitals. Results include total cholesterol (TC), low-density lipoprotein (LDL), very low-density lipoprotein (VLDL), high-density lipoprotein (HDL), and triglycerides (TRIG). Children who also have the AN marker receive additional testing for insulin and glucose. Universal blood cholesterol screening of children identifies children likely to have serious inherited familial hyperlipidemias, such as familial hypercholesterolemia (FH). Children who are at probable risk of FH have LDL values at, or greater than, 190 mg/dL and a positive family history.

### 2.5. Program Procedures

Regional coordinators visit participating classrooms to provide educational sessions on cardiovascular disease, diabetes, and risk factors for other chronic conditions related to lifestyle and inherited conditions. Once complete, CARDIAC consent forms and a descriptive booklet are left with the teachers to distribute to all children and their families. Shortly before the scheduled screening date, regional coordinators collect all returned forms and send them to the main CARDIAC office where they are processed to develop a database, labels, and other support items on screening day. Screening day begins early before classes in a large room—typically the cafeteria or gymnasium. Upon completion of the screening, children receive a token incentive (e.g., bookbag tag, stickers) and additional educational materials. Between 4 to 6 weeks after screening, families receive a health report including screening values, information on how to interpret their findings, and recommendations. Results are also shared with the primary care physician if parent consent is obtained as well as school nurses for followup as appropriate. A toll-free hotline is available for families for questions after they have received screening results. All procedures require at least one parent or legal guardian consent and have been approved by the Institutional Review Board at West Virginia University. Children are not obligated to participate in all screening portions. 

## 3. Results

### 3.1. Participant Characteristics

 Since the project's inception, The CARDIAC Project team has screened 81,156 fifth-grade children. Slightly more than half of these children were female (53.0%). The majority of children were Caucasian (93.2%), 2.9% of the sample was African-American, and 2.3% of children described themselves as biracial. The remaining sample slightly represented Asian (0.4%), Hispanic (0.7%), or “other” (0.5%). 

 Slightly more than half of fifth grade children throughout the state participate in the CARDIAC Project. A sample of 342 parents provided their views of the screening opportunity and identified factors that influenced their decisions to consent (or not consent) their children to participate [12]. Only two differences were found between children who participate in the CARDIAC Project and those who do not. First, the parents of the participating children were more likely to have health insurance than parents of children who did not participate. Participants were also more likely to have a health care provider. Participants did not differ from nonparticipants on any health outcomes (e.g., BMI) or demographic variables (e.g., age, financial status, and gender).

### 3.2. Individual-Level Impact

**Figure 1:**
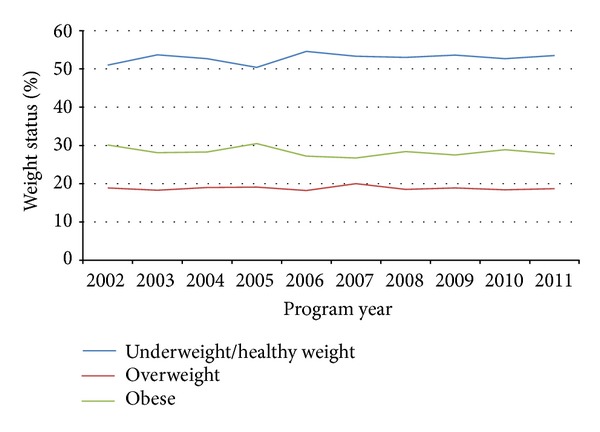
Body composition results in past decade.

**Figure 2:**
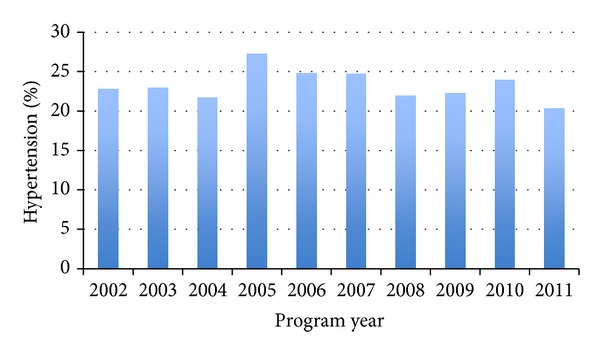
Percentage of sample with hypertension in past decade.

**Figure 3:**
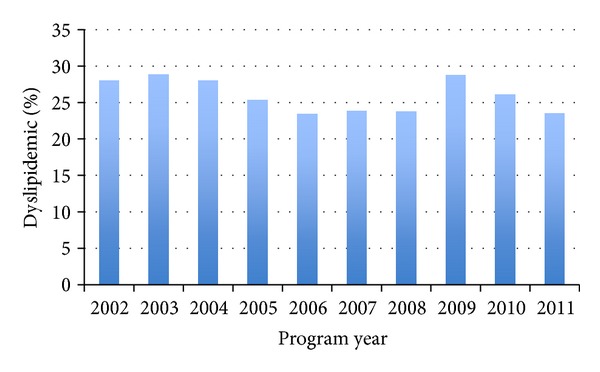
Percentage of sample with abnormal lipid results in past decade.

Children's screening results since the project's inception are provided in Table 1. A closer examination of children's body compositions over the past decade (Figure 1) illustrates a steady prevalence of overweight and obese among screened children. Similar patterns are illustrated in Figures 2 and 3 for children's hypertension and abnormal lipids. When we examine these outcomes across various body compositions, we notice increased risks for those children who are overweight or obese. Comparisons of CARDIAC screening outcomes based on children's body composition illustrate the role of obesity as a hub for other health issues (Table 2). Presence of the AN marker increases from 0.6% of children in the under- or normal weight categories to 2.2% of overweight, 10.2% of obese, and 32.3% of those in the morbidly obese category. Likewise, increases in triglycerides and blood pressure parallel the presence of the AN marker. As expected, HDL cholesterol is inversely related to BMI. Notably there is only a modest increase in LDL from normal weight to overweight, which does not increase in prevalence further as weight status worsens. Only 10–12% of obese youth have hypercholesterolemia and rarely it is severe enough to fulfill criteria for cholesterol-lowering medication. In contrast, there is evidence of progressive insulin resistance as the severity of obesity worsens. 

### 3.3. Family-Level Impact

#### 3.3.1. Screening Impact on Parents' Intention to Change Health Behaviors

Parent telephone interviews conducted between four and six weeks after screening revealed that a portion of parents of children who were identified as having at least one risk factor from the CARDIAC Project would follow up on the screening results. Particularly, 40% of 342 parents of at-risk children had made changes to their children's diets in that short-term period; 34% had modified their children's physical activity opportunities. Twelve percent of the parents of at risk children had made other changes to their children's health care after receiving the health reports from the screening project [12].

#### 3.3.2. Use of an Individualized Approach for Children at Risk and Their Families

The CARDIAC Project encourages healthcare providers throughout WV to monitor at-risk CARDIAC participants' BMI and other health indicators and to prescribe healthy practices to their patients, both children and their families. CARDIAC participants who are at a particularly high risk are referred to a specialized children's lipid clinic for regular intervention and followup. The CARDIAC Intervention Team, along with other experts, developed and led a statewide intervention for overweight/obese children between the ages of 11 and 14 and their caretakers. Three cohorts of the year-long program, Camp NEW (Nutrition, Exercise, and Weight Management), were enrolled and participated in the program between 2008 and 2011. CARDIAC screening participants whose BMI was above the 85th percentile were invited to enroll in the program, where children participated in a two-week summer residential camp and three follow-up family weekends throughout the year. The program included parent educational sessions and one-on-one family counseling throughout the program.

#### 3.3.3. Additional Family Screenings

Approximately 107 of children participating in the project were found to have probable FH. The criteria for diagnosing FH are a total cholesterol value greater than 6.7 mmol/L or LDL-C greater than 4.0 mmol/L in a child who is 16 years old or younger plus DNA-based evidence of an LDL receptor mutation in a first- or second-degree relative. Parents of these children are strongly encouraged to bring their child to one of five children's lipid clinics strategically located around the state; it is recommended that all first-degree relatives likewise have blood cholesterol levels measured and undergo treatment as indicated. Currently, a family-based program focusing on cascade screening of affected probands has been initiated, since it would be expected that half of the child's close relatives would be affected by the same genetic dyslipidemia. To date, 82 family members have been screened to elucidate the family history pathways. Many of these children fulfill national recommendations for consideration of cholesterol lowering medication, if LDL levels remain above acceptable levels following a period of lifestyle modification [13].

### 3.4. Community-Level Impact

#### 3.4.1. Use of Aggregate Data to Inform Actionable Strategies for Change

Aggregate data from the CARDIAC Project is used at the school, county, and state levels to inform decision makers (e.g., educators, administrators, funders, and policy leaders) of the need for targeted interventional strategies. Schools and counties are provided aggregate data on all students screened each year and are encouraged to use the CARDIAC data specific to their children to seek funding and support for increased physical activity opportunities and improved dietary intake in the schools and communities where their children and their families live, learn, work, and play.

#### 3.4.2. Project Development Stimulation

CARDIAC data for specific counties may also be used in project proposals from school systems to increase local efforts that fuel healthy lifestyles. West Virginia On the Move (WVOM) is a statewide, nonprofit organization that promotes a physically active lifestyle throughout the state in three areas: schools, seniors, and communities. Since 2005, their *Schools on the Move* program has awarded 58 grants to schools in 30 WV counties. The *Schools on the Move* program is a collaborative project with the CARDIAC Project. Both programs contribute to the support through the minigrants provided to school personnel for projects including (but not limited to) new walking trails, biking resources, heart rate monitors, and active living programming.

### 3.5. Policy-Level Impact

The breadth and sustainability of the CARDIAC Project have significantly impacted policy and practice within the state and beyond. The unique comprehensive focus on cardiovascular disease and diabetes risk factor screening of more than 10,000 children annually throughout the state contribute to the project's influence. 

#### 3.5.1. West Virginia's Healthy Lifestyle Act of 2005 (HB2816)

 This legislation mandated BMI measurement in schools, as well as an amount of time required for physical education in all grades and a restriction of available sweetened beverages. The CARDIAC Project was named as the mechanism by which the state would meet mandated BMI measurement among school-age children. Members of the CARDIAC team also contributed to other aspects of House Bill 2816, including contributions to writing the bill and expert recommendation. 

#### 3.5.2. Influencing National Youth Screening Guidelines

CARDIAC's goal of offering comprehensive risk factor screening, including a fasting blood draw to all fifth-grade students for whom there is parental consent, provided an opportunity to retrospectively apply targeted National Cholesterol Education Program (NCEP) criteria for screening based on family history of premature heart disease. The purpose of the comparison between targeted versus universal screening among over 20,000 youth was to validate the use of family history in identifying children with severe or genetic hyperlipidemias [14]. The findings demonstrated that targeted (selective) criteria for screening would have missed over one-third (37%) of children with moderate dyslipidemia who warranted consideration of pharmacologic treatment following a trial of lifestyle modification. Subsequent guidelines recommend screening all children at least once during childhood.

### 3.6. Factors Contributing to Program Sustainability

#### 3.6.1. Institutional Review Board (IRB) Packaging for Community Programs

The required components of institutional review form documents can become a barrier to community-based studies especially if unaccompanied by less intimidating informational materials. Communicating the appropriate consent and assent elements in a way that improves health and study literacy should be an essential goal of any community-based program. In an effort to clearly present study elements, a modified consent/assent document was developed for the CARDIAC Project in 1999. Particularly, the approved IRB consent and assent documents consisted of an eight-page booklet. The booklet includes information about what to expect on screening day, the risk factors assessed in the program, and followup after the screening. All required consent elements (e.g., risks, benefits, voluntary participation, and confidentiality) were embedded within a personal letter from the Director and Principal Investigator of the CARDIAC Project.

#### 3.6.2. Joint Agreements with Hospitals, Laboratories, and Schools

 The success of the CARDIAC Project required a growing volunteer workforce and central staff to effectively process children's screening results. Expansion also imposed another goal that CARDIAC team members move beyond detection of risk factors to referral and treatment of documented risks. To effectively meet these expanding aims, the project began to establish joint agreements with local hospitals, laboratories, and schools throughout the state. Over time, agreements have become more formalized. Signed permission by school superintendents is required prior to a screening within the district. School boards are not solicited as that is, at the discretion of the superintendent. Hospitals and laboratory agreements describe the type of documentation and response time following receipt of the blood specimen. Discussions have incorporated elements of participant confidentiality, new online communication including electronic transmission of laboratory results, and better ways to link children's and parents' screening results. 

#### 3.6.3. Enhanced Recruitment Efforts

 Multiple modifications were made to the recruitment procedures in response to findings from an NHLBI-funded grant examining ways to enhance CARDIAC Project participation [12]. First, an educational session for students was created and implemented prior to the screening date. This session complimented the health content areas of instruction that the fifth-grade teachers are required to provide. The session provides an opportunity to discuss the elements of a healthy lifestyle in order to prevent chronic illness such as diabetes and heart disease. 

 A second modification was made to the health report to children and their families after screening. Initial health reports provided the raw screening values for height, weight, BMI, blood pressure, and the lipids. A modified version of the report provided new sections on how to interpret one's screening results (including a visual scale for parents to interpret their children's BMI results), recommendations, and educational facts for physical activity and nutrition. Participants were also asked if they wanted to share their screening results with their primary care provider. This new component was added to help facilitate the followup process between the participant and provider.

## 4. Discussion

The CARDIAC Project has grown from a school-based program in three counties of a rural state to a statewide evidence-based leader in children's chronic disease risk factor screening. Since its inception, more than 80,000 children have received comprehensive cardiovascular risk screening, interventions tailored to their immediate community and/or family, and health education they may apply throughout their lives. Program impact is evident at the individual and family levels based on the number of children screened, the number of children referred for additional services, and the additional services provided for those families who may be affected by a genetic predisposition. 

 While select screening outcomes were maintained over time, others like children's insulin levels rose sharply in the same period. Before we can best interpret these findings, we must understand that only children who presented with the AN marker were screened additionally for insulin and glucose. This limits the generalizability of these findings. With this in mind, we may be witnessing a rise in diabetic risks. These findings may also be the result of advanced screening and analyses methods in corporate laboratories. 

 The CARDIAC Project has contributed to the larger community through its collaborative efforts and the number of small grants funded to change the built environment or add to the course offerings for healthy lifestyle skills building. Community representatives also utilize the screening results as pilot information and leverage for additional grants and other resources coming into their areas. Finally, the policy impact of the project is noticeable at the state and national levels. Very few surveillance programs are able to contribute to the discussion of new cholesterol screening guidelines at the national level. Furthermore, the ability of the program staff to respond to community and state needs has helped position the program as one means of obtaining valuable health assessments for the state. 

 The project has also witnessed an unusual sustainability record over the past 15 years in a state that is underserved and at great risk for many chronic health conditions such as diabetes and heart disease. The CARDIAC Project's growth and success have resulted from an effective balance between being responsive to the needs of rural communities and implementing a national model of innovation in health surveillance and intervention. As needs and evidence changed over the years, so have the procedures, measures, and reporting for the project. The project has also expanded to meet the additional, yet related, needs of the state policy makers and school administration. Together, these individuals have effectively impacted children's health, access to diagnosis and treatment services, and the overall climate and discussions related to childhood obesity and lifestyle behaviors.

## Figures and Tables

**Table 1:** 5th-grade screening results, 1998–2012.

	Year 1	Year 2	Year 3	Year 4	Year 5	Year 6	Year 7	Year 8	Year 9	Year 10	Year 11	Year 12	Year 13	Year 14	Total
	1998-1999	1999-2000	2000-2001	2001-2002	2002-2003	2003-2004	2004-2005	2005-2006	2006-2007	2007-2008	2008-2009	2009-2010	2010-2011	2011-2012	
															
Number of participating counties	3	7	14	27	40	53	55	54	53	55	55	53	50	51	—
Eligible 5th-grade population	781	1.487	3.549	8.495	13.168	18.274	20.854	19.220	17.720	19.778	18.169	18.642	17.845	14.628	192.610
Children screened number (%)	349 (44.7)	709 (47.7)	1.247 (35.1)	3.917 (46.1)	5.973 (45.4)	8.984 (49.2)	9.008 (43.2)	9.257 (48.2)	7.599 (42.9)	7.794 (39.4)	7.816 (43.0)	7.547 (40.5)	6.176 (34.6)	4.780 (32.7)	81.156 (42.1)
Underweight-normal <85th percentile number (% of screened)	188 (55.8)	360 (52.1)	647 (53.3)	2.039 (54.6)	2.948 (51.0)	4.633 (53.7)	4.603 (52.7)	4606 (50.4)	3.878 (54.6)	4.109 (53.3)	4.139 (53.0)	4.039 (53.6)	3.253 (52.7)	2.555 (53.5)	41.999 (52.9)
Overweight BMI ≥ 85th–94th% number (% of screened)	59 (17.5)	141 (20.4)	213 (17.5)	684 (18.3)	1.089 (18.9)	1.576 (18.3)	1.664 (19.0)	1.741 (19.1)	1.293 (18.2)	1.538 (20.0)	1.447 (18.5)	1.422 (18.9)	1.136 (18.4)	892 (18.7)	14.895 (18.8)
Obese BMI ≥ 95th% number (% of screened)	90 (26.7)	190 (27.5)	355 (29.2)	1.017 (27.2)	1.736 (30.1)	2.426 (28.1)	2.469 (28.3)	2.785 (30.5)	1.935 (27.2)	2.060 (26.7)	2.218 (28.4)	2.075 (27.5)	1.781 (28.9)	1.325 (27.8)	22.461 (28.3)
Blood pressure ≥ 95th percentile number (% screened)	69 (24.8)	133 (20.7)	174 (29.7)	823 (23.9)	1.210 (22.8)	1.824 (22.9)	1.757 (21.7)	2.034 (27.2)	1.405 (24.8)	1.565 (24.7)	1.627 (21.9)	1.587 (22.2)	1.470 (23.9)	962 (20.3)	16.640 (23.4)
Child abnormal lipid value (% of FLP sample)	25 (73.5)	21 (77.8)	18 (72.0)	109 (58.0)	214 (28.0)	757 (28.8)	1.173 (28.0)	1.375 (25.3)	1.392 (23.4)	1.451 (23.8)	1.465 (23.7)	1.697 (28.7)	1.458 (26.1)	1.050 (23.5)	12.204 (25.7)
Acanthosis nigricans (AN) number with present marker (%)	—	—	—	111 (3.1)	347 (5.9)	430 (5.0)	378 (4.4)	522 (7.0)	442 (6.9)	498 (7.7)	252 (3.8)	301 (4.1)	248 (4.1)	187 (3.9)	3.716 (5.2)
Child abnormal insulin level (% of AN positive + ordered insulin sample)	—	—	—	19 (11.1)	20 (23.0)	57 (23.9)	56 (30.8)	106 (42.6)	99 (35.4)	126 (40.8)	72 (43.6)	118 (41.0)	162 (42.4)	72 (62.1)	907 (36.8)

**Table 2:** Mean (SD) screening results based on weight status.

	Underweight and normal weight *N* = 24,818	Overweight *N* = 8,958	Obese *N* = 10,375	Morbidly obese *N* = 3,082	ANOVA *P* value
Total cholesterol (mg/dL)	158.18 (26.58)	162.38 (29.29)	166.54^A^ (31.1)	165.81^A^ (31.75)	<0.001
HDL cholesterol (mg/dL)	54.4 (11.97)	49.28 (11.06)	45.05 (10.19)	41.73 (9.28)	<0.001
LDL cholesterol (mg/dL)	89.38 (23.85)	94.84 (26.03)	98.56^A^ (26.96)	98.39^A ^(27.71)	<0.001
Non-HDL cholesterol (mg/dL)	104.1 (25.54)	113.6 (29.04)	122.01 (30.79)	124.68 (31.71)	<0.001
Triglycerides (mg/dL)	73.67 (37.08)	94.13 (53.29)	118.36 (67.96)	133.2 (72.79)	<0.001
Systolic blood pressure (mmHg)	104.43 (10.64)	108.93 (10.68)	112.44 (11.02)	118.82 (12.11)	<0.001
Diastolic blood pressure (mmHg)	66.02 (8.88)	68.63 (8.83)	70.84 (8.99)	74.53 (9.26)	<0.001

^A^indicates no difference in pairwise comparison within weight category; analysis conducted on log-transformed triglycerides.
